# Genome-wide investigation of *AP2/ERF* gene family in the desert legume *Eremosparton songoricum*: Identification, classification, evolution, and expression profiling under drought stress

**DOI:** 10.3389/fpls.2022.885694

**Published:** 2022-08-12

**Authors:** Mingqi Zhao, Yakupjan Haxim, Yuqing Liang, Siqi Qiao, Bei Gao, Daoyuan Zhang, Xiaoshuang Li

**Affiliations:** ^1^State Key Laboratory of Desert and Oasis Ecology, Xinjiang Institute of Ecology and Geography, Chinese Academy of Sciences, Ürümqi, China; ^2^College of Resources and Environment, University of Chinese Academy of Sciences, Beijing, China; ^3^Turpan Eremophytes Botanical Garden, Chinese Academy of Sciences, Turpan, China

**Keywords:** *AP2/ERF* genes, *Eremosparton songoricum*, drought stress, gene structure, gene expression, legume plant

## Abstract

*Eremosparton songoricum* (Litv.) Vass. is a rare leafless legume shrub endemic to central Asia which grows on bare sand. It shows extreme drought tolerance and is being developed as a model organism for investigating morphological, physiological, and molecular adaptations to harsh desert environments. APETALA2/Ethylene Responsive Factor (AP2/ERF) is a large plant transcription factor family that plays important roles in plant responses to various biotic and abiotic stresses and has been extensively studied in several plants. However, our knowledge on the AP2/ERF family in legume species is limited, and no respective study was conducted so far on the desert shrubby legume *E. songoricum*. Here, 153 *AP2/ERF* genes were identified based on the *E. songoricum* genome data. *EsAP2/ERFs* covered *AP2* (24 genes), *DREB* (59 genes), *ERF* (68 genes), and *Soloist* (2 genes) subfamilies, and lacked canonical *RAV* subfamily genes based on the widely used classification method. The *DREB* and *ERF* subfamilies were further divided into A1–A6 and B1–B6 groups, respectively. Protein motifs and exon-intron structures of *EsAP2/ERFs* were also examined, which matched the subfamily/group classification. *Cis*-acting element analysis suggested that *EsAP2/ERF* genes shared many stress- and hormone-related *cis*-regulatory elements. Moreover, the gene numbers and the ratio of each subfamily and the intron-exon structures were systematically compared with other model plants ranging from algae to angiosperms, including ten legumes. Our results supported the view that *AP2* and *ERF* evolved early and already existed in algae, whereas *RAV* and *DREB* began to appear in moss species. Almost all plant *AP2* and *Soloist* genes contained introns, whereas most *DREB* and *ERF* genes did not. The majority of *EsAP2/ERFs* were induced by drought stress based on RNA-seq data, *EsDREBs* were highly induced and had the largest number of differentially expressed genes in response to drought. Eight out of twelve representative *EsAP2/ERFs* were significantly up-regulated as assessed by RT-qPCR. This study provides detailed insights into the classification, gene structure, motifs, chromosome distribution, and gene expression of *AP2/ERF* genes in *E. songoricum* and lays a foundation for better understanding of drought stress tolerance mechanisms in legume plants. Moreover, candidate genes for drought-resistant plant breeding are proposed.

## Introduction

Drought is a major abiotic stress for plants, which can disrupt or prevent seed germination and growth and reduces yield (Lamaoui et al., 2018; Priya et al., 2019). Plants have evolved diverse morphological, biochemical, physiological, and molecular adaptations to changing environments (Valliyodan and Nguyen, 2006). Particularly plants inhabiting harsh environments, such as deserts or highly saline habitats, have evolved strategies to adapt to such stress conditions. *Eremosparton songoricum* (Litv.) Vass. is a rare and extremely drought-tolerant legume shrub distributed in Central Asia; its distribution is fragmented, and it occurs around Balkhash Lake in Kazakhstan and the Gurbantunggut Desert in China (Zhang et al., 2008; Liu et al., 2011). *Eremosparton songoricum* grows naturally on bare sand and can tolerate multiple extreme environmental conditions, including drought, high and low temperatures, and UV radiation (Liu et al., 2016, 2017). *Eremosparton songoricum* has evolved various morphological adaptation strategies to adapt to severe desert water deficit; for example, its leaves are extremely reduced into photosynthetic branches to reduce water loss, and its roots grow horizontally to facilitate cloning propagation so as to optimize water utilization (Liu et al., 2010; Shi et al., 2010). Meanwhile, rapid asexual reproduction expands the inhabited area, which can ameliorate the soil and fix the sand, thus helping protect the habitat. Therefore, *E. songoricum* is a promising taxon for desertification control and environmental protection (Shi et al., 2010; Liu et al., 2013, 2016). Previous studies at physiological and molecular levels have also shown that *E. songoricum* is a good model legume for studying the molecular mechanisms of extreme drought stress and identifying genes associated with drought tolerance for crop breeding (Li et al., 2012, 2013, 2014, 2016).

Transcription factors (TFs) play critical roles in gene expression regulation networks in response to various environmental stresses. TFs control downstream genes by binding *cis*-acting elements in the promoter regions of target genes. Previous research on plant stress tolerance improved through genetic transformation of TF genes has yielded several achievements (Xu et al., 2011). APETALA2/ethylene responsive element binding proteins (AP2/ERF) is one of the largest TF families, and is a key regulator of plant responses to various stresses (Gu et al., 2017; Baillo et al., 2019; Feng et al., 2020). The AP2/ERF family can be divided into five subfamilies, i.e., AP2, DREB, ERF, RAV, and Soloist, according to the type and number of AP2 and B3 conserved domains. *AP2* subfamily genes have two AP2 domains that are involved in flower and seed development (Jofuku et al., 1994; Ripoll et al., 2011); *ERF* and *DREB* subfamilies have only one AP2 domain, which is the largest gene member among the AP2/ERF family and plays key roles in plant biotic and abiotic stresses (Mizoi et al., 2012). *ERF* subfamily genes play important roles in plant biotic stress and pathogen responses (Park et al., 2001; Gutterson and Reuber, 2004). *DREB* subfamily genes are dominant regulators of plant abiotic stress and are prominent during osmotic and cold stress (Agarwal et al., 2006). The *RAV* subfamily comprises one AP2 domain and one B3 domain, which are involved in floral induction, bud outgrowth, leaf senescence, and responses to pathogen infections and abiotic stresses (Matias-Hernandez et al., 2014). *Soloist* is a small subfamily of genes possessing a single AP2 domain, but their sequences are highly divergent from those of other *AP2/ERF* genes (Lakhwani et al., 2016), and they function in plant salt and pathogen responses (Giri et al., 2014; Sun et al., 2016). Many *AP2/ERF* genes have been isolated from various plants and have been widely used to improve crop stress tolerance (Agarwal et al., 2017; Sarkar et al., 2019).

With the development of high-throughput sequencing, more than 700 plant genomes have been released (Kersey, 2019), which has greatly promoted the identification and functional analysis of *AP2/ERF* genes. AP2/ERF TFs have been identified on a genome-wide scale in model plants such as *Arabidopsis thaliana* and *Oryza sativa* (Nakano et al., 2006; Ahmed et al., 2021) and in non-model plants including *Vitis vinifera*, *Brassica rapa*, *Brassica oleracea*, *Sesamum indicum*, *Dimocarpus longan*, and *Populus trichocarpa* (Zhuang et al., 2008; Licausi et al., 2010; Song et al., 2013; Dossa et al., 2016; Li H. et al., 2017; Zhang et al., 2020). In legumes, the *AP2/ERF* family has been identified in *Glycine* max (Zhang et al., 2008; Jiang et al., 2020), *Medicago truncatula* (Shu et al., 2016), *Cicer arietinum*, *Cajanus cajan* (Agarwal et al., 2016), and *Lotus corniculatus* (Sun et al., 2014; Cao et al., 2021); however, these results stem from only a small proportion of the thousands of legume species. In addition to the identification results published previously, identification results of AP2/ERF in 36 legumes can be found in the Plant Transcription Factor Database (Plant TFDB 5.0). To date, *AP2/ERF* gene mining in legume plants is rather limited and not particularly detailed; thus, further research on legume plants and more accurate and detailed identification is required.

We recently sequenced the genome of *E. songoricum* (China National GeneBank [CNGB], project ID: CNP0002419; 555.67 Mb; 26,442 genes; eight chromosomes). In this study, we first identified AP2/ERF TFs in *E. songoricum* based on genome data. In total, 153 *EsAP2/ERF*s, including four subfamilies (AP2, DREB, ERF, and Soloist), were identified, and their detailed classification, gene structure and motif analysis, chromosomal distribution, *cis*-element analysis, and gene expression patterns under drought stress were examined. Moreover, to better understand the evolution and expansion of *AP2/ERFs*, we identified and classified the *AP2/ERF* family in 20 other representative plant species, ranging from algae to angiosperms, including nine other legume plants, to perform a comparative analysis, including subfamily number, ratio, and gene structure characteristics. This is the first study to report genome-wide identification of *AP2/ERF* family genes in legume shrubs. Our results provide insight into the drought stress mechanisms in legume plants, and we propose candidate genes for drought-resistant plant breeding.

## Materials and methods

### Plant materials and drought stress treatments

*Eremosparton songoricum* seeds were collected from the Gurbantunggut Desert Xinjiang province, China (88°24′67′′E, 45°58′11′′N). Seeds were soaked in 98% (v/v) sulfuric acid for 10–15 min to break physical dormancy, after which they were washed with ddH_2_O and sown on moist filter paper. Seeds were germinated and grown in covered petri dishes under controlled conditions (25°C, 12 h darkness, 100 mol m^–2^ s^–1^ light intensity, 60% relative humidity). To each Petri dish (150 mm) containing seeds, 15 mL ddH_2_O was added every 2 days.

Two-week-old seedlings with similar primary root lengths were transferred to a culture box containing 400 mL PEG6000 (20% w/v) for the drought treatment. For each time point (0, 6, 12, 24, and 36 h), 10 whole plants were harvested, pooled, and rapidly frozen in liquid nitrogen for RT-qPCR assay, and samples at 0 h served as controls.

### Identification and classification of *AP2/ERF* genes

A total of 26,442 genes were obtained from *E. songoricum* genome data. The genome was submitted to CNGB^1^ under the project ID: CNP0002419. A Hidden Markov model (HMM) scan with PF00847 (AP2 domain) and PF02362 (B3 domain) was used to mine the *AP2/ERF* gene sequences in *E. songoricum* and the other 22 plant genomes (Mistry et al., 2021). The profiles were queried using the HMM search command of TBtools-HMMER software (e-value cutoff 1 × 10^–3^) (Chen et al., 2020). The other 22 plant genomes were downloaded from the NCBI genome^2^ and Phytozome 13.0^3^ (Goodstein et al., 2011). The AP2 domain of AP2/ERFs with a length of approximately 60 amino acids was considered a full-length AP2 domain protein. All predicted peptide sequences contained at least one complete AP2 domain.

### Conserved motif detection and molecular weight, isoelectric point, and subcellular localization prediction in *Eremosparton songoricum*

The conserved motifs in the AP2/ERF protein sequences were analyzed using the online tool Multiple Expectation Maximization for Motif Elicitation (MEME, version 5.3.3)^4^ with default parameters (Bailey et al., 2015): only motifs with an e-value under 0.1 were retained for further analysis. Conserved motifs were visualized using the Evolview V3 web tool^5^ (Subramanian et al., 2019). Molecular weights and isoelectric points of EsAP2/ERFs were predicted using SwissProt-Expasy.^6^ Subcellular localization analysis was performed using Busca^7^ (Savojardo et al., 2018).

### Multiple sequence alignment and phylogenetic analyses

We downloaded 175 Arabidopsis AP2/ERF predicted amino acid sequences from the Plant Transcription Factor Database (PlantTFDB V5.0)^8^ (Tian et al., 2019). The protein sequences of 153 EsAP2/ERFs from *E. songoricum* and 175 AtAP2/ERFs from *A. thaliana* were used to construct phylogenetic trees. Multiple sequence alignment was performed using ClustalW (Thompson et al., 1994), phylogenetic trees were constructed by the neighbor-joining method (with 1,000 bootstrap replicates) using MEGA 11, and evolutionary distances were computed using Poisson correction with pairwise deletion (Tamura et al., 2021). The phylogenetic trees were visualized using Evolview web (Subramanian et al., 2019).

### Chromosomal locations, intron-exon structures, and *cis*-element analysis of *AP2/ERF* genes

Gene duplication type and Ka/Ks were calculated using the MCSCAN module in TBtools (Chen et al., 2020). Information on chromosome distribution (including chromosome length and the starting positions) was retrieved from the local genome annotation and visualized using Circos module in TBtools (Chen et al., 2020). The intron-exon structures of the *AP2/ERF* genes were assessed based on the annotation file of 21 plant genomes and were visualized using Evolview web (Subramanian et al., 2019). The 3,000-bp region upstream of the transcriptional start site of 153 EsAP2/ERF genes was used for *cis*-element site analysis using the PlantCARE website.^9^

### Protein-protein interaction network

The STRING database^10^ was used to assemble the protein–protein interaction (PPI) networks (Szklarczyk et al., 2021). Based on the annotation of transcriptome and identification in genome, a total of 153 AP2/ERFs and 1,276 other TFs in *E. songoricum* were used to predict PPI network. The minimum required interaction score was set to medium confidence (0.6). Cytoscape (version 3.8) was used for image processing.

### Transcriptome-based expression analysis

Transcriptome data (CNGB, CNP0003150) was used to analysis the expression abundance of 153 *EsAP2/ERF* genes under drought stress at 0, 6, 12, 24, and 36 h. Three biological replicates were performed. Differentially expressed genes (DEGs) were determined using FDR < 0.05 and |log2fold change| > 1. TBtools was used to make Heatmap access genes’ relative expression levels.

### RT-qPCR assay and data analyses

Total RNA was isolated using the MiniBEST plant RNA kit (Takara, Kyoto, Japan), and first-strand cDNA was synthesized using the PrimeScript™ RT reagent kit (Takara). Twelve EsAP2/ERF genes representative of different subfamilies were selected to explore gene expression patterns. RT-qPCR primers were designed using Primer Premier 5.0, and primer specificities were tested by executing a BLASTn search against local *E. songoricum* genome data. Primer specificity was further assessed using melting curve analysis of the RT-qPCR. All primers used for RT-qPCR are listed in Supplementary Table 1. RT-qPCR experiments were carried out using the CFX96 Real-Time PCR Detection System (Bio-Rad, Hercules, CA, United States) with the SYBR *Premix Ex Taq*™ kit (Takara). Three biological replicates and three technical replicates of each biological replicate were used for all samples. The RT-qPCR protocol was as follows: initial denaturation at 95°C for 30 s and 40 cycles of 94°C for 5 s and 60–62°C for 30 s. Gene expression levels were calculated relative to the 0 h timepoint using the 2^–ΔΔ*Ct*^ method (Livak and Schmittgen, 2001). The *EsEF* gene was used to normalize RT-qPCR results (Li et al., 2012).

Statistical analyses were performed, and plots were produced using Graph Pad Prism (version 9.0), and all data were analyzed using one-way analysis of variance (ANOVA) at 95% confidence level. Differences were tested using the least significant difference multiple comparison test.

## Results

### Genome-wide identification and classification of *AP2/ERFs* in *Eremosparton songoricum*

**FIGURE 1:**
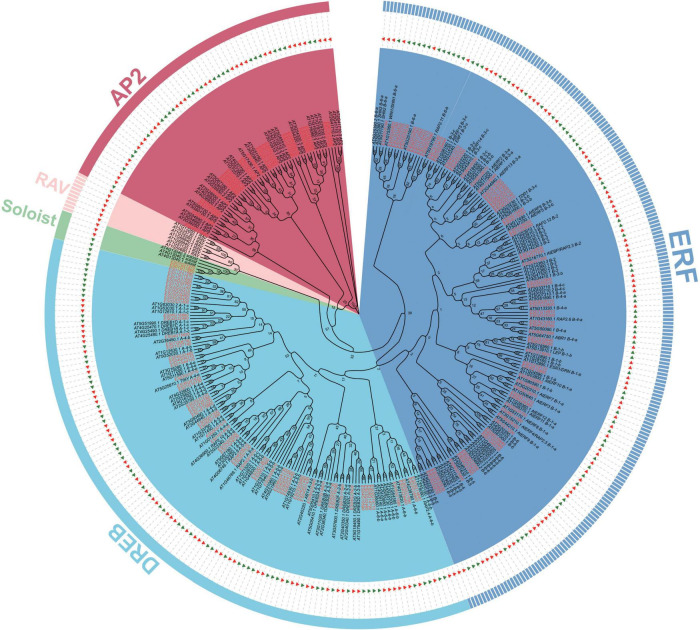
Phylogenetic analysis of *AP2/ERF* genes in *Eremosparton songoricum*. A neighbor-joining phylogenetic tree was constructed using MEGA 11 with AP2 domains of AtAP2/ERFs and EsAP2/ERFs. The numbers of bootstrap values were based on 1,000 iterations. AP2, DREB, ERF, RAV, Soloist subfamilies are grouped together as indicated in red, cyanine, blue, pink, and green, respectively. EsAP2/ERF are indicated by green triangles and red text, and AtAP2/ERFs are indicated by red triangles and black text.

Based on the *E. songoricum* genome, 153 *AP2/ERF* genes were identified. A total of 175 AtAP2/ERFs and 153 EsAP2/ERFs were used to construct a phylogenetic tree to classify EsAP2/ERFs. Phylogenetic analyses divided 153 EsAP2/ERF members into four subfamilies: AP2, DREB, ERF, Soloist; RAV subfamily members were not found, based on the genome data (Figure 1). The classification results were confirmed by counting the number of conserved domains (AP2 and B3 domains). Among the confirmed family members, 24 EsAP2/ERFs were assigned to the cluster of the AP2 subfamily based on the phylogenetic tree and presence of the tandemly repeated double AP2 domain (Figure 1). A total of 127 EsAP2/ERFs clustered in the ERF (68 TFs) and DREB (59 TFs) subfamilies, which contained a single AP2 domain. The small Soloist subfamily included two members in *E. songoricum* (Figure 1). In summary, we identified 24 AP2s, 59 DREBs, 68 ERFs, and two Soloists based on the *E. songoricum* genome. Considering that some studies reported that transcriptional factors with only one B3 are also considered to belong to the RAV subfamily (Zhao et al., 2017; Chen et al., 2021), we performed a BLASTp search against the *E. songoricum* genome using the full length of seven Arabidopsis RAVs and 13 soybean RAVs (8 GmRAV only had one B3 domain) as queries, and three hits with high scores were obtained (coverage ≥ 90%; e-value < 1^–10^; Supplementary Table 2). The predicted protein lengths of 153 EsAP2/ERF members ranged from 110 to 757 amino acids, molecular weight ranged from 12.7 to 86.5 kD, and the protein isoelectric point ranged from 4.4 to 9.8. Most EsAP2/ERFs (88%) were predicted to be located in the nucleus, and two Soloist members were predicted to be localized in the chloroplast (Supplementary Table 3).

### Gene number and ratio comparison analysis of *AP2/ERFs* in various plants

**FIGURE 2:**
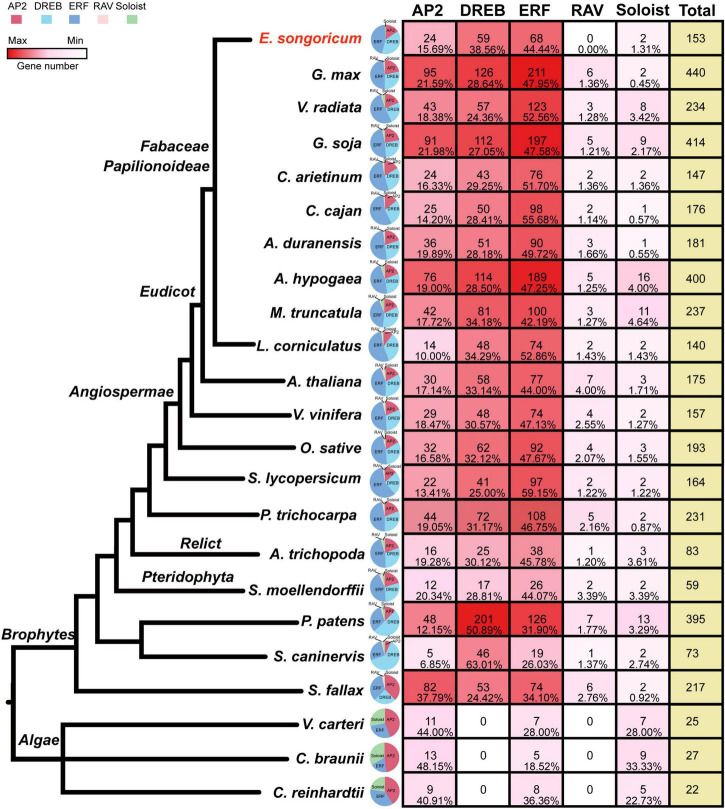
Classification and gene number/proportion analysis of *AP2/ERF* family genes in 23 plant species. HMM scan with PF00847 (AP2 domain) and PF02362 (B3 domain) were used to identify AP2/ERF family in 23 species, and the subfamily classification of 22 species were performed by MEGA 11, with 175 AP2/ERF TFs from *Arabidopsis thaliana* as a reference. The quantity of gene members in different species is shown as a heatmap (see color bar), the maximum is indicated in dark red, the minimum (0) is shown in white. A pie chart shows the ratio of each family, and AP2, DREB, ERF, RAV, Soloist subfamily are shown in red, cyanine, blue, pink, and green, respectively; the numbers and percentage of each subfamily member were shown. The species tree was based on previous literature (Zeng et al., 2014, 2017; Huang et al., 2016).

To better understand the evolution of the AP2/ERF family, in addition to *E. songoricum* and *A. thaliana*, we also identified the *AP2/ERF* family in 21 other representative plants ranging from algae and early land plants to angiosperms, including 10 legume species. We found that in three algae (*V. carteri*, *C. braunii*, and *C. reinhardtii*), only *AP2*, *ERF*, and *Soloist* subfamilies were present. The *DREB* and *RAV* subfamily began to occur in mosses (Figure 2), and *DREB* and *ERF* subfamilies were consistently represented with numerous members among different plant species, whereas the RAV subfamily retained only few members. The Soloist subfamily represented a large proportion in algae, whereas in land plants it comprised few members.

Among ten legume plants, the other nine representative legume species all contained five subfamilies of AP2/ERF, except for *E. songoricum*, which lacks a canonical *RAV*, according to the classic classification method. The number of *AP2/ERFs* in the ten legumes ranged from 140 (*L. corniculatus*) to 440 (*G. max*). The proportion of *ERF* subfamily genes was higher than that of *DREBs*, which exceeded 40% in the ten legume plants. The *DREB* subfamily in *E. songoricum* was represented at the highest proportion (38.56%) among the ten legumes, followed by *L. corniculatus* (34.29%), and *M. truncatula* (34.18%). The ratio of the *RAV* subfamily was <2%, and the numbers of genes ranged from 2 to 6 (Figure 2), *Soloist* gene numbers varied among legumes, ranging from 1 (*C. cajan* and *A. duranensis*) to 16 (*A. hypogaea*).

### Detailed classification of the EsDREB subfamily

**FIGURE 3:**
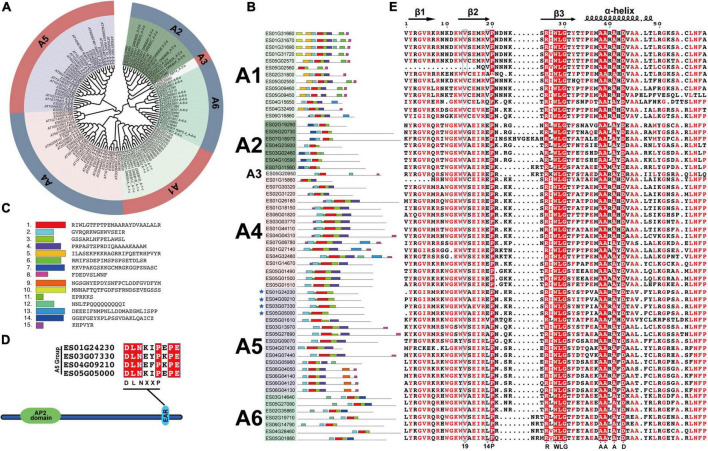
Classification and sequence analysis of EsDREBs subfamily. The phylogenetic tree was constructed based on AP2 domains of AtDREBs and EsDREBs using MEGA 11 with the neighbor-joining method. 15 motifs were observed using the MEME webtool. The AP2 domains of 59 EsDREBs were used to perform multiple sequence alignment. **(A)** Phylogenetic tree of EsDREB subfamily. **(B)** Motif distributions in A1–A6 group of EsDREBs. **(C)** Amino acid sequence of each motif. Genes with EAR motif are indicated by stars **(D)** EsDREB genes with EAR motif. **(E)** Multiple sequence alignment of AP2 domains of EsDREBs.

The DREB subfamily plays key roles in plant abiotic stress responses and can be further divided into A1–A6 groups. In the present study, 59 EsDREBs were classified into 6 groups by constructing a phylogenetic tree with DREBs of *A. thaliana*. There were 13, 7, 1, 16, 15, and 7 members in the A1–A6 groups of EsDREBs, respectively (Figure 3A). Fifteen conserved motifs were detected in the protein sequence of the EsDREBs (Figures 3B,C). Motifs 1, 2, and 3 were composed of the AP2 domain, of which motif 1 contained the β3 sheet and α-helix, motif 2 contained β1 and β2 sheets of the AP2 domain; motif 3 represented the C-terminal of the AP2 domain. Other motifs were divergent among different groups, such as motif 10 and motif 5 which occurred only in the A1 group; motif 7 was specific to the A2 group, whereas the EAR repressor motif was detected in the C-terminus of four A-5 type EsDREBs (Figure 3D). Multiple sequence alignment analysis of the AP2 domain showed that EsDREBs shared significant amino acid similarity; 9 out of the 59 amino acids in AP2 domains, including the residues 20-P, 26-R, 28-W, 29-L, 30-G, 38-A, 39-A, 41-A, and 43-D, were completely conserved among all EsDREBs (Figure 3D). In addition, consistent with other plant DREBs, the 14th V residue was completely conserved among all EsDREB members except ES05G02560 which lacks some amino acids in the N-terminal region, while the 19th amino acid had multiple patterns, including E/V/L/A/Q (Figure 3E).

### Detailed classification of the *EsERF* subfamily

**FIGURE 4:**
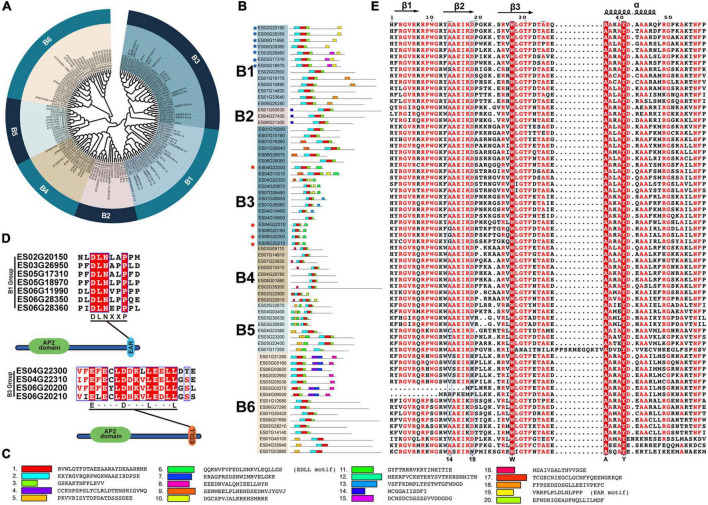
Classification and sequence analysis of EsERFs subfamily. The phylogenetic tree was constructed based on AP2 domain sequence of AtERFs and EsERFs using MEGA 11 with neighbor-joining; 25 motifs were discovered using MEME online tools. AP2 domains were used for multiple sequence alignment. **(A)** Phylogenetic tree of the EsERF subfamily. **(B)** Motif distribution in B1–B6 groups of EsERFs, genes with EAR or EDLL motif are indicated by blue and red stars, respectively. **(C)** Amino acid sequence of each motif. **(D)** Genes with EAR motif and EDLL motifs. **(E)** Multiple sequence alignment of AP2 domains of EsERFs.

EsERF subfamily can be further classified into six groups (Figure 4A). A total of 13, 3, 19, 9, 8, and 16 EsDREBs were identified for B1–B6, respectively (Figures 4A,B). In total, 20 motifs in 68 EsERF proteins were observed, of which motif 2 contained β1 and β2 sheets, motif 1 was composed of β3 and an α-helix, and motif 3 was the C-terminus of the AP2 domain. Furthermore, ERFs in the same group mostly shared a similar motif composition in addition to the AP2 domain; for example, motif 14 was shared among the B2 group, motif 16 was unique to the B4 group, and motif 5 was only present in the B5 group. The transcription repressor and activator motifs EAR (motif 19) and EDLL (motif 6) occurred at the C-end of B1 and B3 EsDREBs, respectively, and four B3 EsDREBs had EDLL motifs, which were distributed in seven B1 EsDREB members (Figures 4B–D). Three out of the 59 amino acid residues, including 27-W, 37-A and 41-Y, which occurred in the β3 sheet and α-helix of all EsERFs, were completely conserved (Figure 4E). The 14th and 19th conserved amino acids were diverse among ERFs, and the patterns of the 14th and 19th position of AP2 domains were A14 and D19 in most EsERFs, while the 14th position also showed a G/V/I/T/C pattern, and the 19th position was changed to H or N in some EsERFs. The AEIR located between the 14th and 19th position was the same in B1–B5, while the four amino acids changed to SEIR or AEIR/K in B6 group EsERFs (Figure 4E).

### Gene structure analysis of *AP2*/*ERFs* in *Eremosparton songoricum* and other plants

**FIGURE 5:**
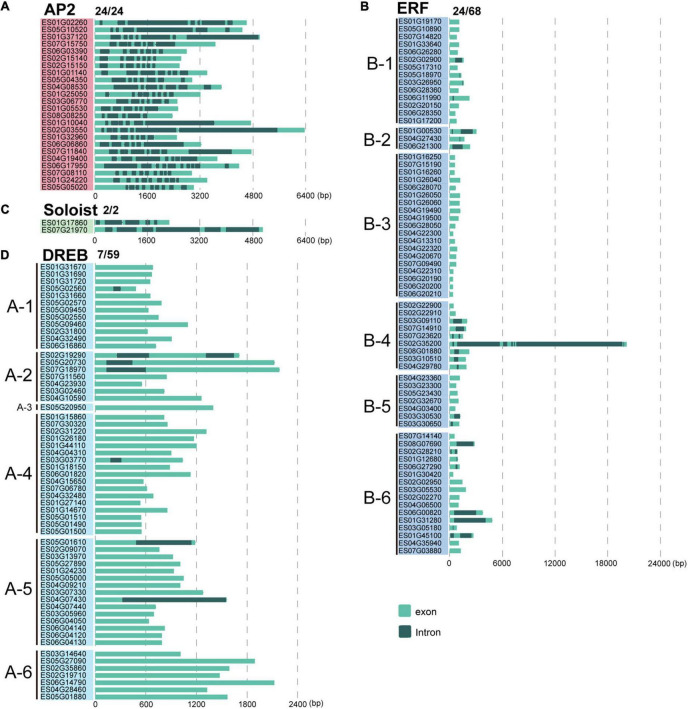
Gene structure analysis of 153 EsAP2/ERF genes. Intron-exon structures were constructed by gene annotation. Panels **(A–D)** show AP2, ERF, Soloist, and DREB subfamilies, respectively. Light green and gray indicate exons and introns, respectively.

To gain insights into gene structural diversity, we examined the exon and intron organization of *EsAP2/ERF* genes. In *E. songoricum*, the proportions of genes with introns in the *DREB*, *ERF*, *AP2*, and *Soloist* subfamilies were 11.9, 35.3, 100, and 100%, respectively. All members of *EsAP2* (24/24) and *EsSoloist* (2/2) had five to ten introns in the *AP2* subfamily and five to six in the *Soloists* subfamily (Figure 5). For the *ERF* subfamily, 24 of 68 *EsERFs* had introns; among them, 19 *EsERFs* had only a single intron, four *EsERFs* had two introns, and one *EsERF* (*ES02G35200*) belonging to the B4 group had seven introns, which also had the longest gene length. For the *DREB* subfamily, only 7 out of 59 *EsDREB* members had introns (*N* = 1–2); 6 of 7 *EsDREBs* only had a single intron, and the remaining one had two introns (Figure 5).

**FIGURE 6:**
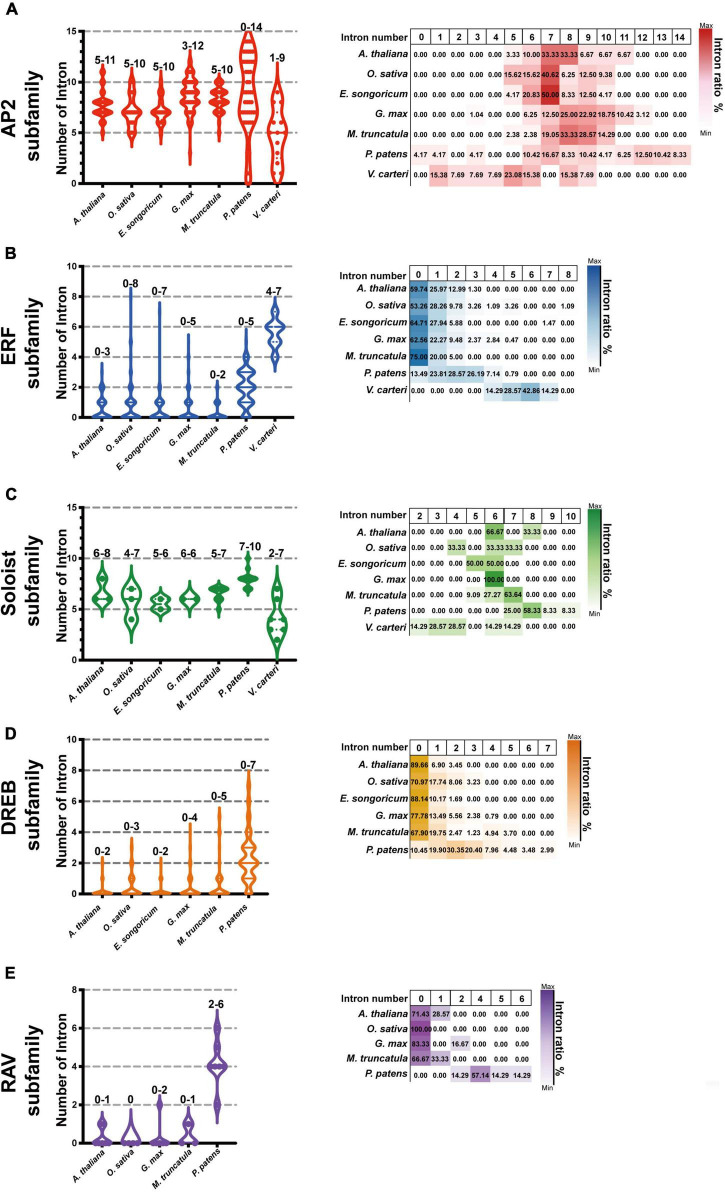
Intron-exon number analysis of EsAP2/ERFs in seven plant species. The genome annotation of seven species including *G. max* and *M. truncatula*, *Arabidopsis*, *O. sativa*, *P. patens*, and *V. carteri* were used to investigate AP2/ERF family intron distribution. Graph pad prism 9 was used to produce box and violin plots; 153 EsAP2/ERF, 440 GmAP2/ERF, 237 MtAP2/ERF, 175 AtAP2/ERF, 193 OsAP2/ERF, 395 PpAP2/ERF, and 25 VcAP2/ERF genes were used for intron-exon distribution analysis. Panels **(A–C)** show intron numbers of AP2, ERF, and Soloist subfamily members of introns of seven plant species. The DREB and RAV subfamilies have not evolved in *V. carteri*, and *Eremosparton songoricum* does not contain the canonical RAV subfamily; thus, panel **(D)** shown are intron numbers and proportions of DREB subfamily from six species (excluding *V. carteri*). Panel **(E)** intron numbers of five species and proportions in the RAV subfamily (excluding *V. carteri* and *E. songoricum*). The intron number range is indicated on top of each column. The violin plots show intron member distribution, and the heatmap (right) shows gene proportions with different intron numbers as indicated by coloration.

We further selected six model plants, including *G. max*, *M. truncatula, A. thaliana, O. sativa, P. patens, and V. carteri*, to perform intron-exon distribution comparisons of *AP2/ERFs* (Supplementary Figures 1–6). Similar to *E. songoricum*, almost all genes belonging to the *AP2* and *Soloist* subfamilies in other plant species had introns, while most *DREB* and *ERF* genes were without intron (Figure 6 and Supplementary Figures 1–6). Overall, the intron number distribution of five subfamilies in algae (*V. carteri*) and moss (*P. patens*) was more dispersed than in angiosperms. Specifically, for the *AP2* subfamily, except for *P. patens* (4.17% of *PpAP2s* were intronless), the other six species all had introns (mainly seven to nine) (Figure 6A); however, regarding the ERF subfamily, most ERFs in angiosperm plants lack introns, and the intron number centered at 2 (Figure 6B); a similar intron-exon distribution pattern was found in *DREB* subfamilies (Figure 6D). For *Soloist* subfamilies, except for *V. carteri* (14.29%), *Soloists* in other species all had introns (mainly six to seven; Figure 6C). A large majority of RAVs in angiosperms were intronless, whereas *PpRAVs* had two to six introns.

### Chromosomal distribution and duplication analysis of *EsAP2*/*ERF*

**FIGURE 7:**
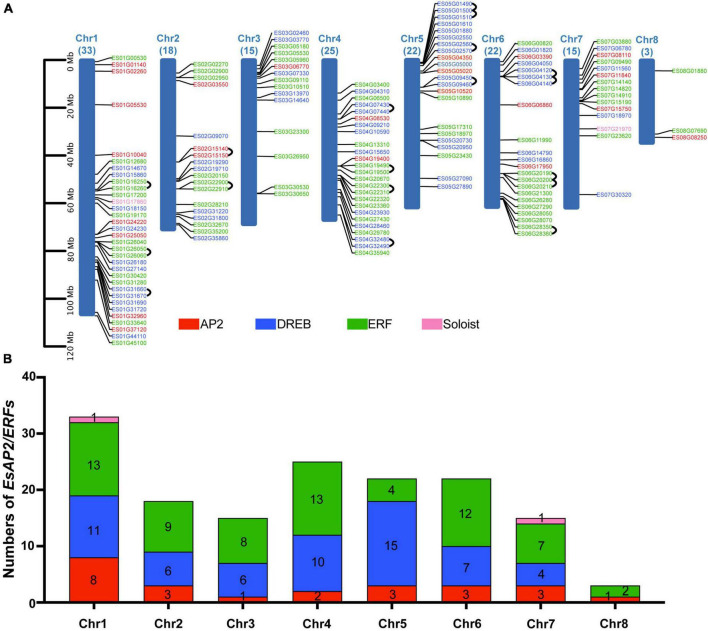
EsAP2/ERF gene distribution and tandem duplication, in eight chromosomes of *Eremosparton songoricum*. BLASTp and gene location information was used to determine gene duplication. **(A)** Chromosome distribution analysis of EsAP2/ERFs, chromosome size is indicated by relative length. The total gene numbers of EsAP2/ERFs are shown on the top of each chromosome. AP2, DREB, ERF, and Soloist genes are indicated in red, blue, green, and pink, respectively. Black loops indicate duplication gene link pairs. **(B)** Distribution numbers of four subfamily genes of EsAP2/ERFs in eight chromosomes.

Chromosome mapping analysis indicated that 153 *AP2/ERF* genes were unevenly mapped on eight chromosomes in *E. songoricum*. Each chromosome contained at least three *EsAP2/ERF* genes. Chromosome 1 contained the largest number (33 genes) of *EsAP2/ERF* genes, followed by chromosomes 4 (25 genes), 5 (22 genes), and 6 (22 genes), whereas chromosome 8 contained only three *EsAP2/ERFs* with two *ERFs* and one *AP2* gene (Figure 7A). Members of the *AP2* and *ERF* subfamilies were widely distributed across all chromosomes. *DREB* genes did not occur on chromosome 8, whereas two Soloist genes were located on chromosomes 1 and 7. Chromosome 1 had 13 *ERFs*, 11 *DREBs*, 8 *AP2s*, and one *Soloist*; chromosome 5 had the largest number of DREB genes (15); chromosomes 1 and 4 had the largest number of *ERFs* (13) (Figure 7B). In order to elucidate the duplication mechanism of the *EsAP2/ERF* family, the gene replication types were analyzed using MCScanX, and the results are shown in Supplementary Table 4. A total of 150 duplication events were found, including 71 segmental or WGD (Whole Genome Duplication), 46 dispersed, 27 tandem, and six proximal duplication events. Furthermore, 15 link gene pairs were found on chromosomes 1, 2, 4, 5, and 6 (Figure 7A). The Ka/Ks values of the 15 link gene pairs were all <1, indicating that they evolved under the pressure of purifying selection (Supplementary Table 5).

### The *cis*-regulatory elements and protein-protein interaction analysis of *EsAP2/ERFs*

Transcription factors play key roles in regulating gene expression by interacting with *cis*-acting elements (Nakashima et al., 2009). To understand the putative function of *EsAP2/ERF* genes in plant stress responses and the regulatory relationship with plant hormones, we analyzed *cis*-elements of 3,000 bp of promoter regions of 153 *EsAP2/ERF* genes. A total of 116 *cis*-elements were found, including six unnamed and 110 reported elements that were involved in distinct biological functions (Supplementary Table 6). We selected 36 classic previously reported *cis*-elements (Chai and Subudhi, 2016; Shi et al., 2019; Li et al., 2021) belonging to 22 categories and divided them into two major functional categories, i.e., phytohormone-responsive and abiotic or biotic stress-related (Supplementary Figure 7 and Supplementary Tables 7, 8). The 22 categories occurred 4,496 times in the promoter region of 153 *EsAP2/ERF* genes, among which MYB, MYC, and ABRE elements were the most enriched (Supplementary Figure 7). Specifically, 99% of the *EsAP2/ERF* genes contained MYB and MYC binding sites that appeared 1,085 and 620 times, representing 37 and 21% of the abiotic and biotic stress-related *cis*-elements, respectively (Supplementary Table 7). The ABRE and ERE, involved in ABA and ET responses, were found 541 and 260 times in 110 and 107 *EsAP2/ERF* genes, respectively, and accounted for 52% of the phytohormone-responsive genes (Supplementary Table 7). Moreover, GA-responsive elements (GARE, P-box, and TATC-box), JA-responsive elements (CGTCA motif, TGACG motif), SA-responsive elements (TCA-element), and auxin-responsive elements (TGA, AuxRR) were present in 122, 110, 102, and 76 EsAP2/ERF genes, respectively (Supplementary Figure 7).

To identify potential interacting proteins with the AP2/ERF transcription factors, a protein - protein interaction (PPI) network was generated with the STRING database (Szklarczyk et al., 2021). A total of 49 EsAP2/ERFs including 23 EsERFs, 19 EsDREBs, 6 EsAP2s, one EsSoloist interacted with 160 other TFs in the network (Supplementary Figure 8 and Supplementary Table 9), in which EsDREBs had the largest number of interacted TFs, then followed by EsERFs. EsAP2/ERFs mainly interacted with WRKYs (17), HSFs (14), MYBs (11), STZs (9), and ABFs (8). The A2 type of DREB (ES02G19290) directly interacted with other 20 TFs, such as ABI5 and ABFs. AP2 (ES04G19400) and Soloist (ES01G17860) only interacted with members of EsAP2/ERFs.

### Expression patterns of *EsAP2/ERF* genes in response to drought stress

**FIGURE 8:**
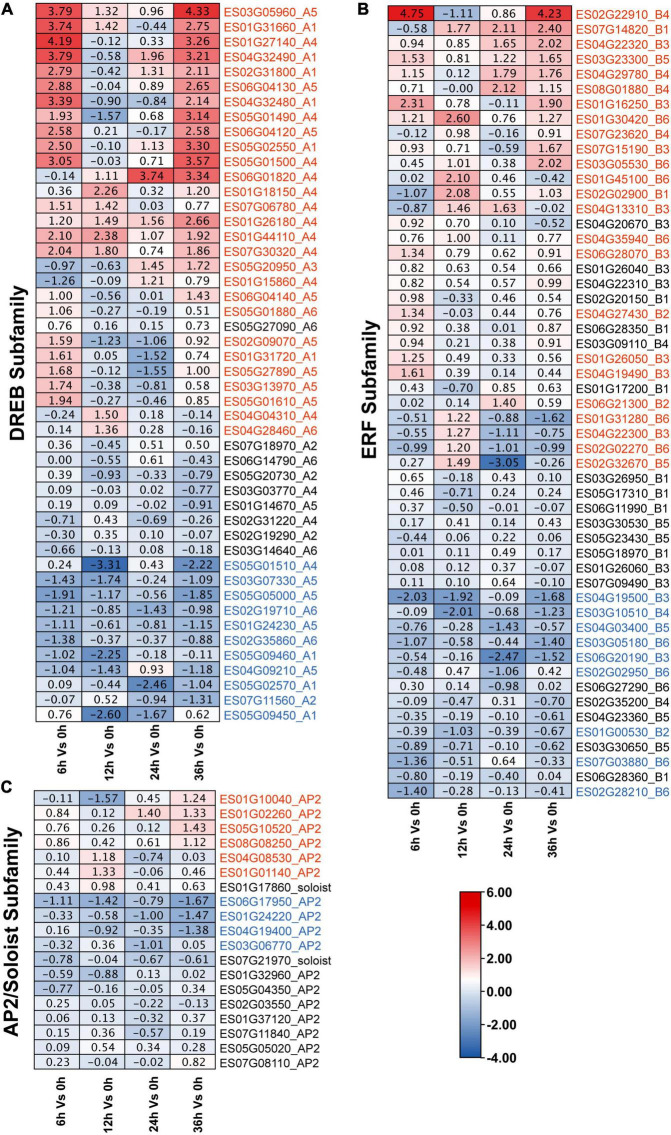
Expression profiles of 120 *EsAP2/ERF* genes response to drought stress based on RNA-seq data. Expression profiles (in |log2fold change| values) of the **(A)** *DREB*; **(B)** *ERF*; and **(C)** *AP2* and *Soloist* subfamily, respectively. Up-regulated genes were marked by red, down-regulated genes were marked by blue.

To further determine the involvement of *EsAP2/ERF* genes in response to drought stress, we analyzed the expression pattern of *EsAP2/ERF* based on transcriptome data (CNP0003150). As a result, a total of 120 *EsAP2/ERF* genes were quantified by mapping reads among 153 *EsAP2/ERFs* (120/153, 78.43%), which contained 53 *ERFs*, 48 *DREBs*, 17 *AP2s*, and 2 *Soloists.* Eighty-one differentially expressed *EsAP2/ERFs* genes included 32 *ERFs* (32/53, 60.4%), 39 *DREBs* (39/48, 81.2%) and 10 *AP2s* (10/17, 58.8%). Differentially expressed *EsAP2/ERFs* were mainly distributed at 6 and 36 h, included 37 and 34 genes, respectively, and notably more *EsAP2/ERF* genes were up-regulated than down-regulated (75/32) (Supplementary Figure 9). Differentially expressed *EsDREBs* included 28 up-regulated and 11 down-regulated genes, respectively (Figure 8A). Up-regulated *DREB* genes consisted of A1, A4, and A5 group, most of genes significantly up-regulated at 6 and 36 h, among them, A4 (*ES01G27140*) and A5 (*ES03G05960*) had the highest expression with more than 16-fold (converted by |Log2 FC|) increase compared with that at 0 h. Differentially expressed *EsERF*s included 23 up-regulated and nine down-regulated genes, respectively (Figure 8B), of which *ES02G22910* (B4 type of *ERF*) had the highest expression with more than 16-fold (converted by |Log2 FC|) increase compared with that at 0 h. *EsSoloist* and *EsAP2* subfamily genes were slight induced after drought stress (Figure 8C).

**FIGURE 9:**
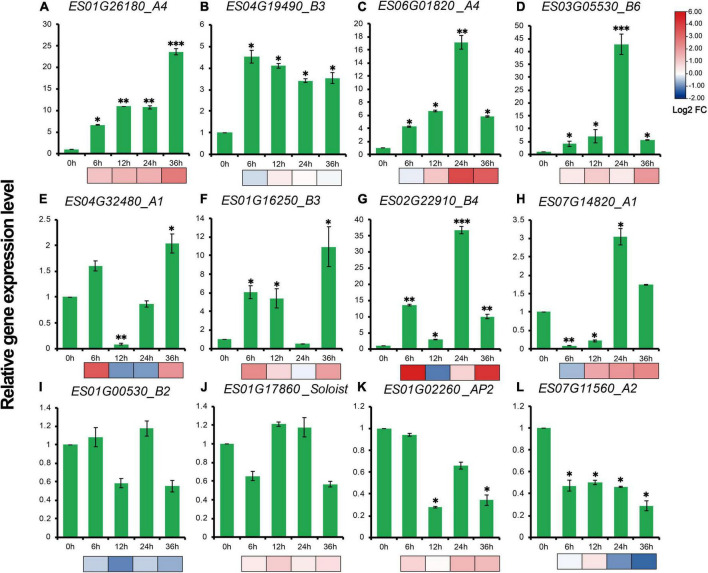
**(A–L)** Gene expression levels of 12 *EsAP2/ERF* genes under drought treatment. The gene expression levels were calculated relative to 0 h using the 2^–ΔΔ*Ct*^ method. Shown are the mean values ± SE of three replicates, and the significance level relative to controls is *P < 0.05; ^**^P < 0.01; ^***^P < 0.001.

RT-qPCR results showed that expression profiles of 12 genes, representative of different family members of *EsAP2/ERFs* including five *DREB*s, five *ERF*s, one *AP2* and one *Soloist* demonstrated diverse gene expression patterns during drought stress treatment. The expression patterns of most selected genes were consistent with the transcriptome results (Figure 9). Eight out of twelve genes were strongly up-regulated in drought stress-treated *E. songoricum*, which highly elevated and reached the peak at 24 or 36 h (Figures 9A–H). Similar like RNA-Seq results, *ES03G05530* and *ES02G22910* also were strongly induced with drought stress and increased almost 40-fold compared with that at 0 h (Figures 9D,G). Two genes including B2 type of ERF (*ES01G00530*) and *Soloist* (*ES01G17860*) were slightly changed (Figures 9I,J), and two genes (*ES01G02260* and *ES07G11560*) exhibited a decline pattern after drought stress (Figures 9K,L).

## Discussion

### Detailed identification and classification of *EsAP2/ERFs* in a desert legume

The *AP2/ERF* gene family is one of the largest groups of TFs in plants, and it is characterized by at least one AP2 domain, which plays important roles in plant development and stress responses (Sakuma et al., 2002; Nakano et al., 2006; Agarwal et al., 2017). In the current study, genome-wide analysis of the *AP2/ERF* gene family from the desert legume *E. songoricum* was conducted, and 153 putative *AP2/ERF* genes were identified. These 153 genes covered the *AP2*, *ERF*, *Soloist*, and *DREB* subfamilies based on domain numbers and sequence similarities. The classic *RAV* gene nomenclature is characterized by the presence of a C-terminal B3 domain and N-terminal AP2 domain (Kagaya et al., 1999). In the present study, we were unable to identify a canonical RAV protein containing both B3 and AP2 domains; however, the other nine legume plants all had canonical *RAV* genes (*N* = 2–6; Figure 2). Previous studies on soybean and rice introduced a classification method with BLASTp searching using AtRAVs protein sequence as query, and genes with only one B3 domain were also considered to be the RAV genes (Zhao et al., 2017; Chen et al., 2021), it is worth noting that these GmRAVs showed different expression patterns under abiotic stresses compared with canonical GmRAVs, implying that they may have different functions. Using this method, we obtained three hits that were probably RAV proteins (Supplementary Table 4); however, further evidence is needed to confirm whether those candidates were EsRAVs. The classification results obtained from the phylogenetic tree and conserved domain numbers were in accordance with the motif and amino acid composition patterns, suggesting that the conserved motifs and amino acid sites can be helpful in classifying the specific subfamily/group of AP2/ERF (Shu et al., 2016; Li X. et al., 2017, Li et al., 2018). Moreover, we found that genes belonging to the same subfamily or specific group shared similar intron-exon organization, which has also been reported in other species (Song et al., 2016; Zhang et al., 2022), indicating that intron-exon structure can also be a useful basis for AP2/ERF family classification.

### Origination and evolution of *AP2/ERFs*

To explore the origin and evolution of the *AP2/ERF* gene family in plants, we identified *AP2/ERF* family genes in 22 other representative plant species ranging from algae to land plants and compared the gene numbers and structures across these 21 plants. Consistent with previous studies, *AP2* and *ERF* already existed in algae, whereas *RAV* and *DREB* began to appear in mosses (Mizoi et al., 2012; Song et al., 2016). Our results also supported the phenomenon that *DREB* and *ERF* occurred at a large proportion, whereas *Soloist* and *RAV* were small subfamilies with very few genes in land plants (Agarwal et al., 2016; Shu et al., 2016; Li X. et al., 2017, Li et al., 2018). In the 10 legume species, the number of *AP2/ERFs* varied from 140 to 440, and *G. max* had the largest number of *AP2/ERF* genes, followed by *G. soja* and *A. hypogaea*, which can be explained by independent whole-genome duplications at the species level (Liu et al., 2020; Zhao et al., 2021). Notably, the number of *DREB* genes in *E. songoricum* accounted for the highest proportion among the ten legume species, which can be partially explained by the adaptation of *E. songoricum* to harsh desert environments.

It is known that ancestral species have intron-rich genes, and most plant species experienced extensive loss or insertion of introns due to selective pressure (Roy and Penny, 2007; Rogozin et al., 2012). Low intron gain rates and intron number reduction are common in eukaryotic evolution (Roy and Penny, 2006, 2007). In the current study, we selected seven representative species ranging from algae and land plants to compare *AP2/ERF* gene structures. Our results showed that almost all *AP2* and *Soloist* subfamily genes of *E. songoricum* and other plant species had introns, whereas most *DREB*, *ERF*, and *RAV* genes were intronless (Figure 6). Angiosperms such as Arabidopsis, *E. songoricum*, and *O. sativa* shared the same intron-exon structures, whereas the number of introns in algae and moss was more variable than in angiosperms. These results are consistent with the intro-exon evolution patterns of *AP2/ERF* genes of various higher species (Dossa et al., 2016; Zeng et al., 2016; Zhang et al., 2020; Ahmed et al., 2021; Cao et al., 2021).

### *AP2/ERF* family genes play important roles in *Eremosparton songoricum* stress responses

*AP2/ERF* genes play important roles in plant abiotic and biotic stress responses, especially in the *DREB* subfamily which exerts important effects under different abiotic stress conditions, including drought, heat, and salt stress (Sakuma et al., 2002; Shi et al., 2019). *Eremosparton songoricum* is an extremely drought-tolerant legume species; hence, it is a good material for stress-tolerance gene isolation (Li et al., 2014). Cis element analysis showed that all *EsAP2/ERF* genes were enriched in ABRE, MYB, and MYC *cis*-elements, suggesting that *EsAP2/ERF* genes may participate in the regulation of abiotic and biotic stress responses and plant hormone transduction pathways. Classification results of the *EsAP2/ERF* gene family showed that *DREB* genes had the largest proportion, compared to the other nine legume plants, indicating the evolutionary expansion of *DREBs* for adaptation to harsh desert environments. We previously cloned a *DREB2* gene from *E. songoricum*, which was associated with strong resistance to drought, salinity, cold, and heat (Li et al., 2014, 2016). Moreover, *EsDREBs* had the largest number of differentially expressed genes and were highly induced in response to drought (39/48, 81.3%), in which 28 *EsDREBs* genes were significantly upregulated in response to drought stress, *ES01G27140* and *ES03G05960* were strongly increased by more than 16-fold compared to normal condition (Figure 8A). Meanwhile, *EsERFs* also remarkably increased after drought stress (Figures 8B,C, 9), indicating that *EsDREBs*/*EsERFs* play important roles in *E. songoricum* response to drought stress and they are good stress tolerance genes for further functional verification.

## Data availability statement

The datasets presented in this study can be found in online repositories. The names of the repository/repositories and accession number(s) can be found in the article/Supplementary material.

## Author contributions

XL conceived the ideas and designed the study. MZ collected and analyzed the data. SQ performed the experiments. MZ and YL wrote the manuscript. XL, DZ, YH, and BG revised the manuscript. All authors contributed to manuscript revision, read, and approved the submitted version.

## Footnotes

1
https://db.cngb.org/

2
https://www.ncbi.nlm.nih.gov/genome/

3
https://phytozome-next.jgi.doe.gov/

4
https://meme-suite.org/meme/

5
https://evolgenius.info

6
https://web.expasy.org/compute_pi/

7
http://busca.biocomp.unibo.it/

8
http://planttfdb.gao-lab.org/

9
http://bioinformatics.psb.ugent.be/webtools/plantcare/html/

10
http://string-db.org
