# Supplementary material for: Genome-wide investigation of AP2/ERF gene family in the desert legume Eremosparton songoricum: Identification, classification, evolution, and expression profiling under drought stress
Source: Front Plant Sci. 2022 Aug 12;13:885694. doi: 10.3389/fpls.2022.885694 (PMC9413063; doi:10.3389/fpls.2022.885694)
Supplement: Supplementary file 1 [file Data_Sheet_1.docx]

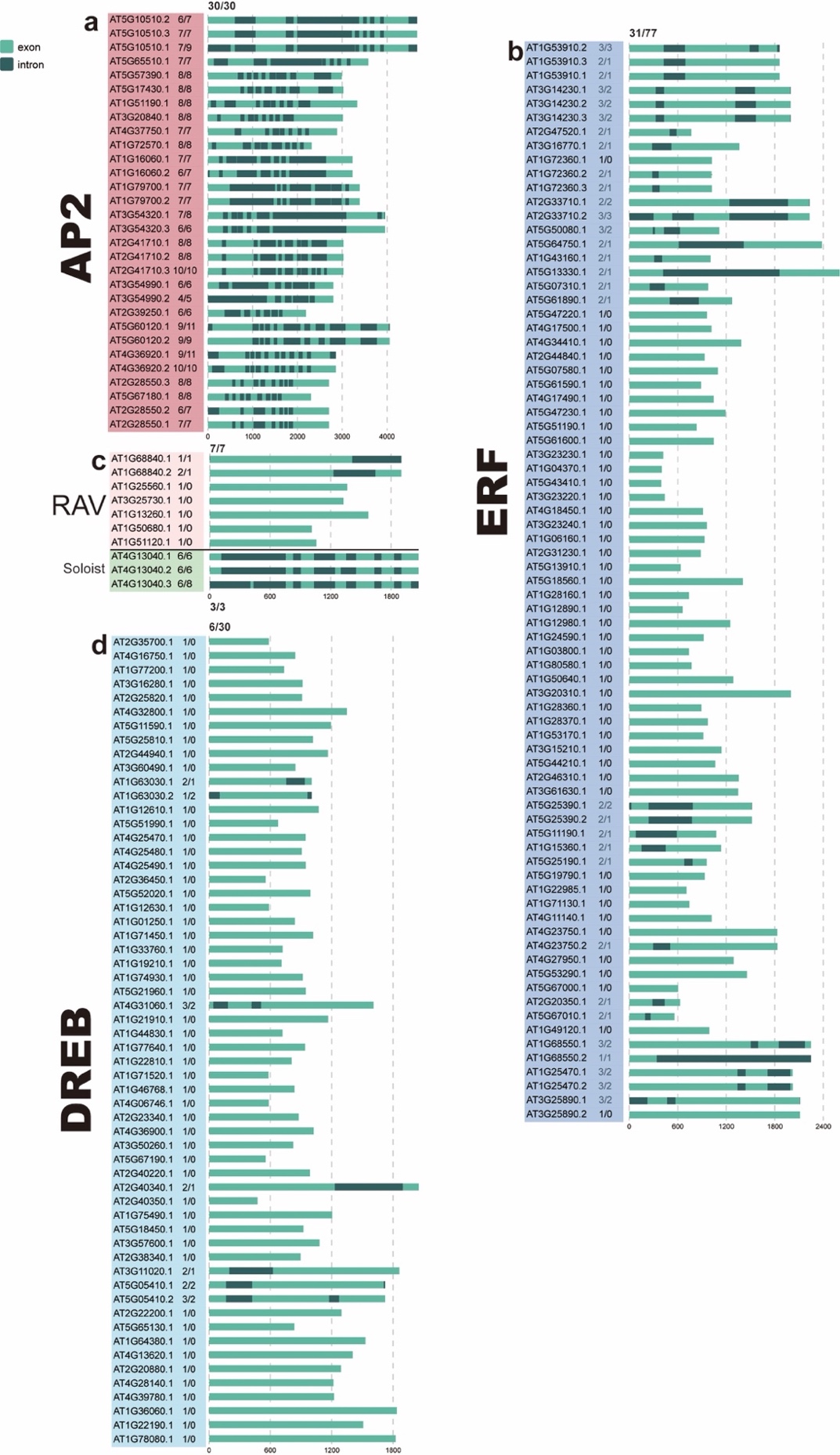


**FIGURE S1 | *AP2/ERF* genes structure in *A. thaliana*.** Based on genome annotation file, the evolview website was used to show *AtAP2/ERF* gene structure. The red, pink, green, blue, and cyanine represent AP2, RAV, Soloist, ERF, and DREB, respectively. The light green and ink green was exon and intron, respectively. This ratio mean the proportion between members containing introns and their all subfamilies.


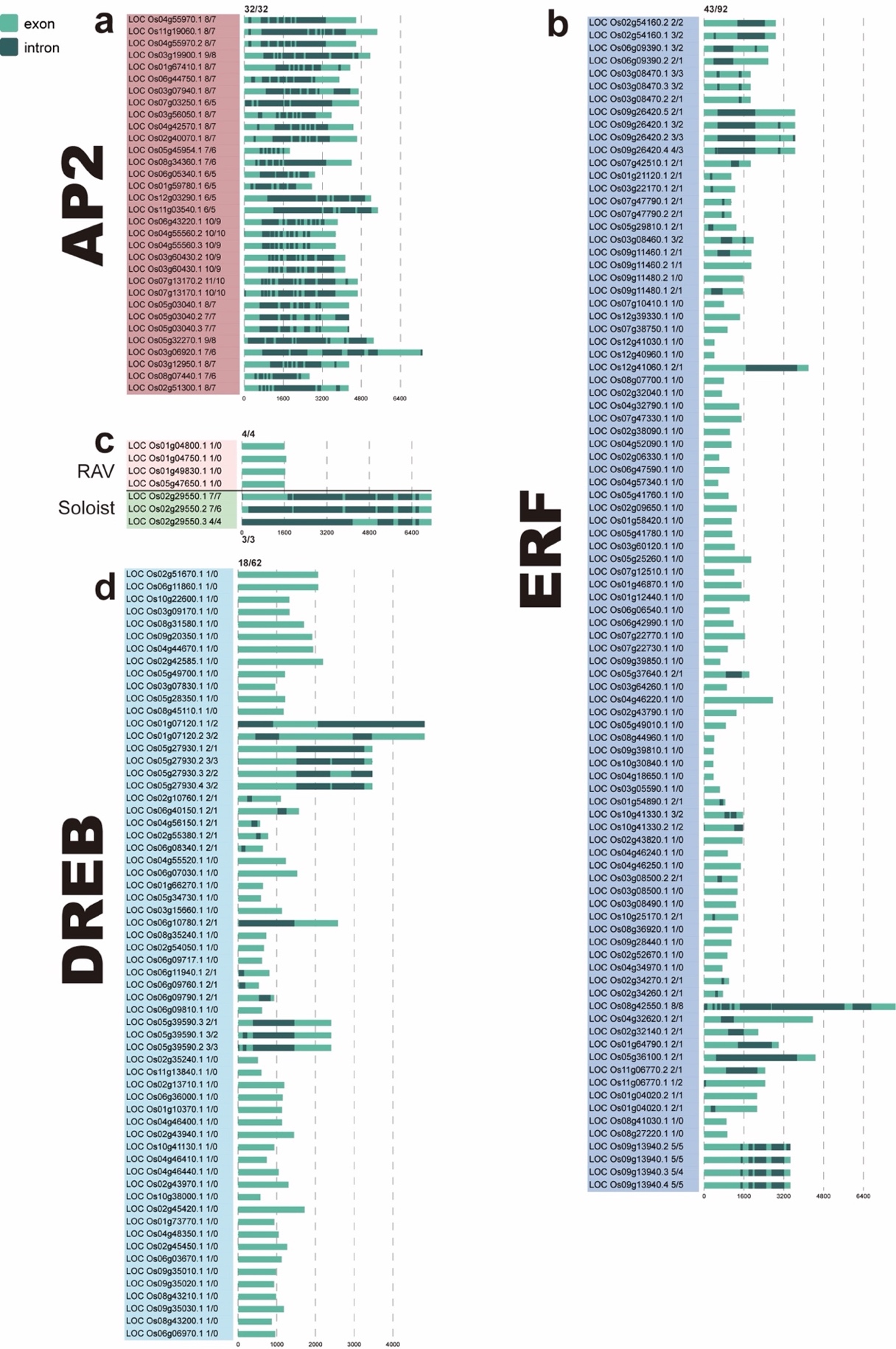


**FIGURE S2 | *AP2/ERF* genes structure in *O. sativa.*** Based on genome annotation file, the evolview website was used to show *OsAP2/ERF* gene structure. The red, pink, green, blue, and cyanine represent AP2, RAV, Soloist, ERF, and DREB, respectively. The light green and ink green was exon and intron, respectively. This ratio mean the proportion between members containing introns and their all subfamilies.

*
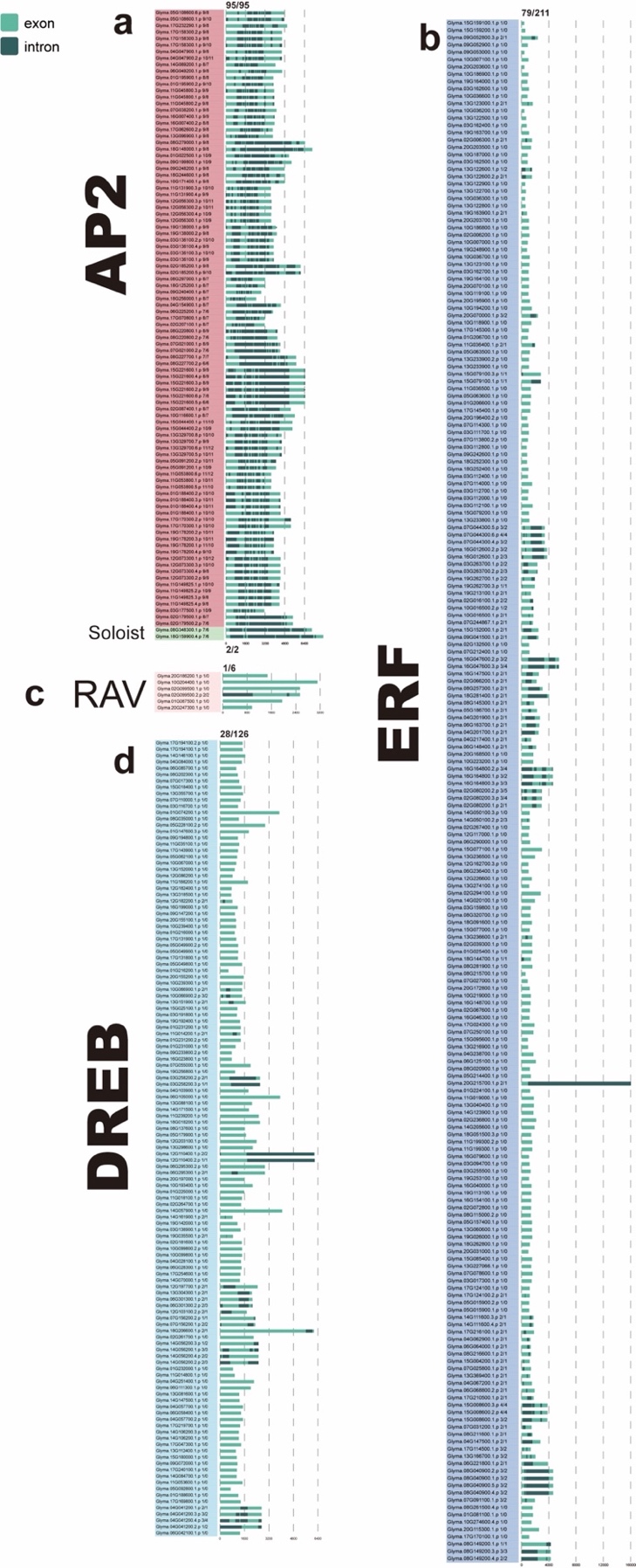
*

**FIGURE S3 | *AP2/ERF* genes structure in *G. max.*** Based on genome annotation file, the evolview website was used to show *GmAP2/ERF* gene structure. The red, pink, green, blue, and cyanine represent AP2, RAV, Soloist, ERF, and DREB, respectively. The light green and ink green was exon and intron, respectively. This ratio mean the proportion between members containing introns and their all subfamilies.


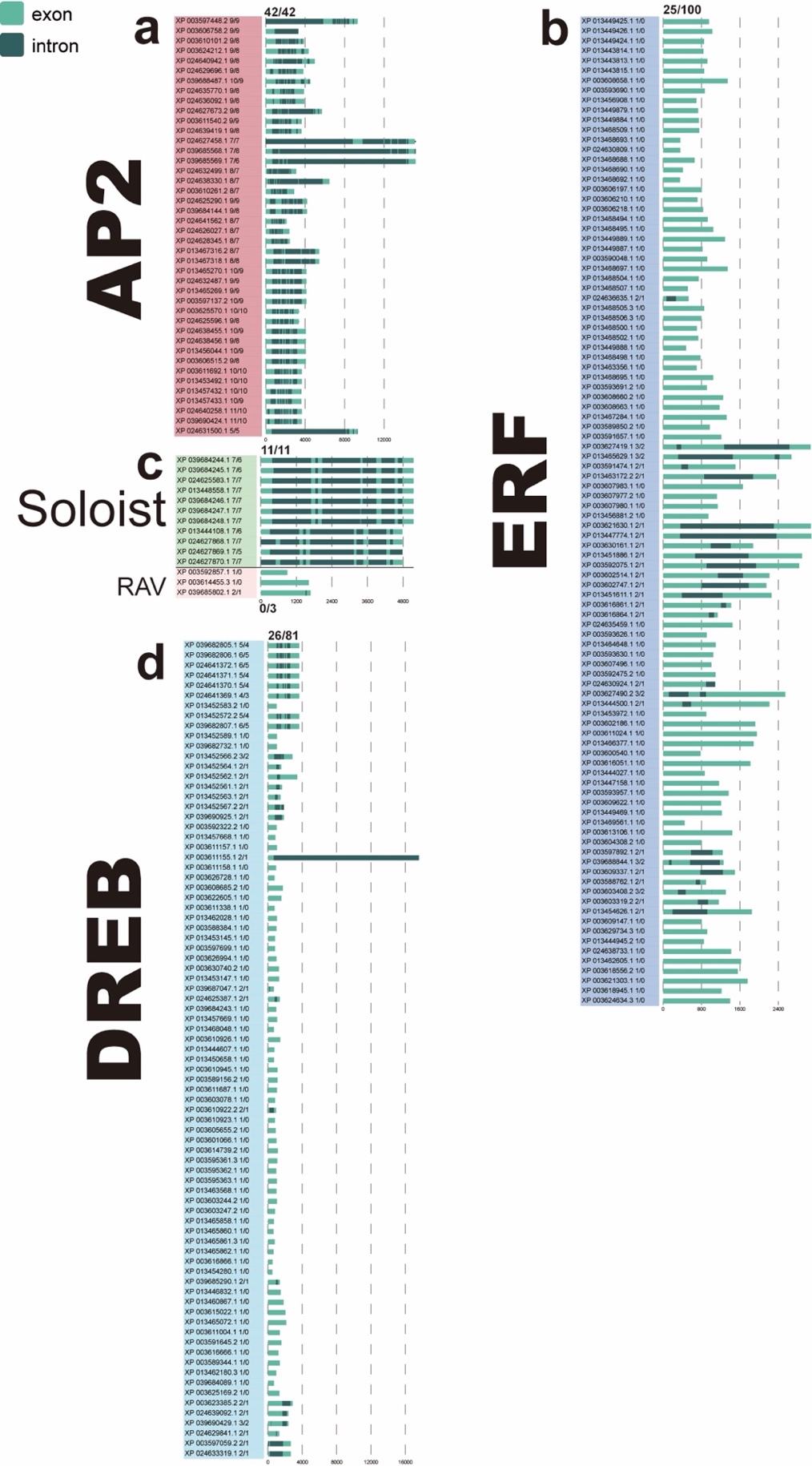


**FIGURE S4 | *AP2/ERF* genes structure in *M. truncatula.*** Based on genome annotation file, the evolview website was used to show *MtAP2/ERF* gene structure. The red, pink, green, blue, and cyanine represent AP2, RAV, Soloist, ERF, and DREB, respectively. The light green and ink green was exon and intron, respectively. This ratio mean the proportion between members containing introns and their all subfamilies.


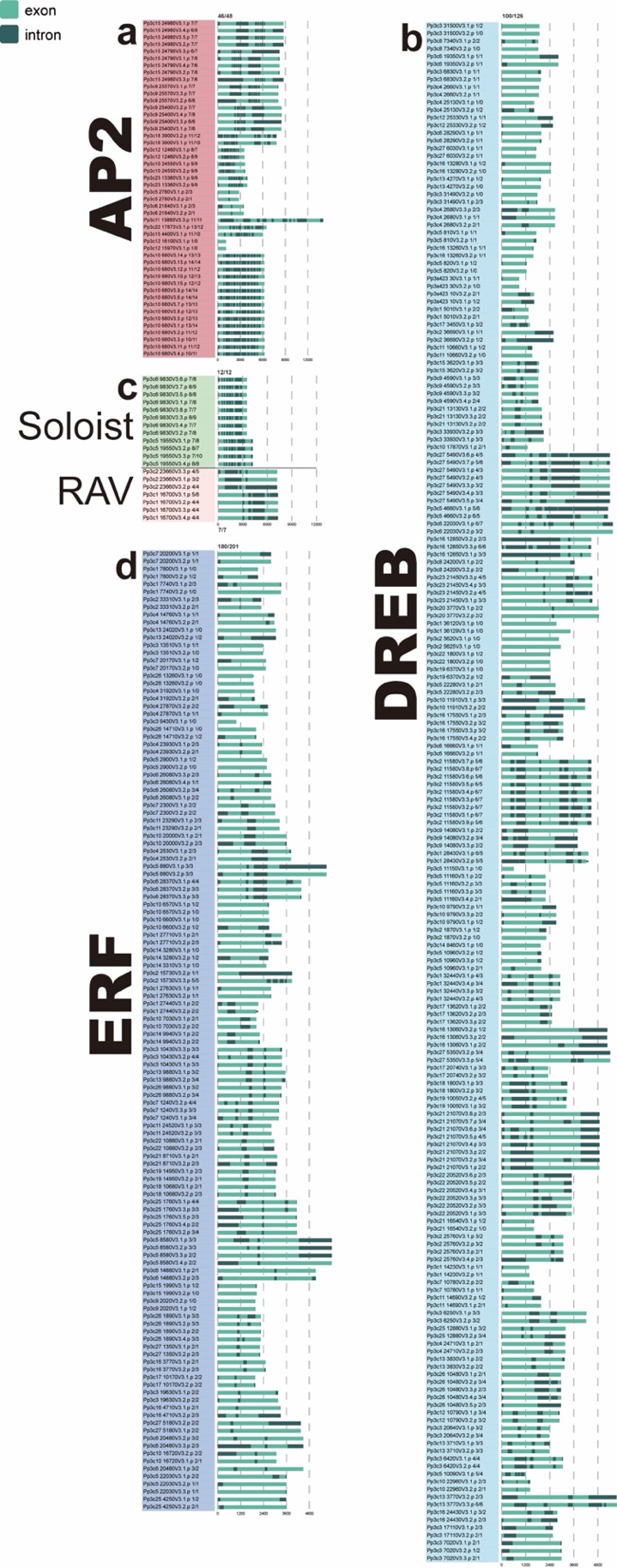


**FIGURE S5 | *AP2/ERF* genes structure in *P. patent.*** Based on genome annotation file, the evolview website was used to show *PpAP2/ERF* gene structure. The red, pink, green, blue, and cyanine represent AP2, RAV, Soloist, ERF, and DREB, respectively. The light green and ink green was exon and intron, respectively. This ratio mean the proportion between members containing introns and their all subfamilies.


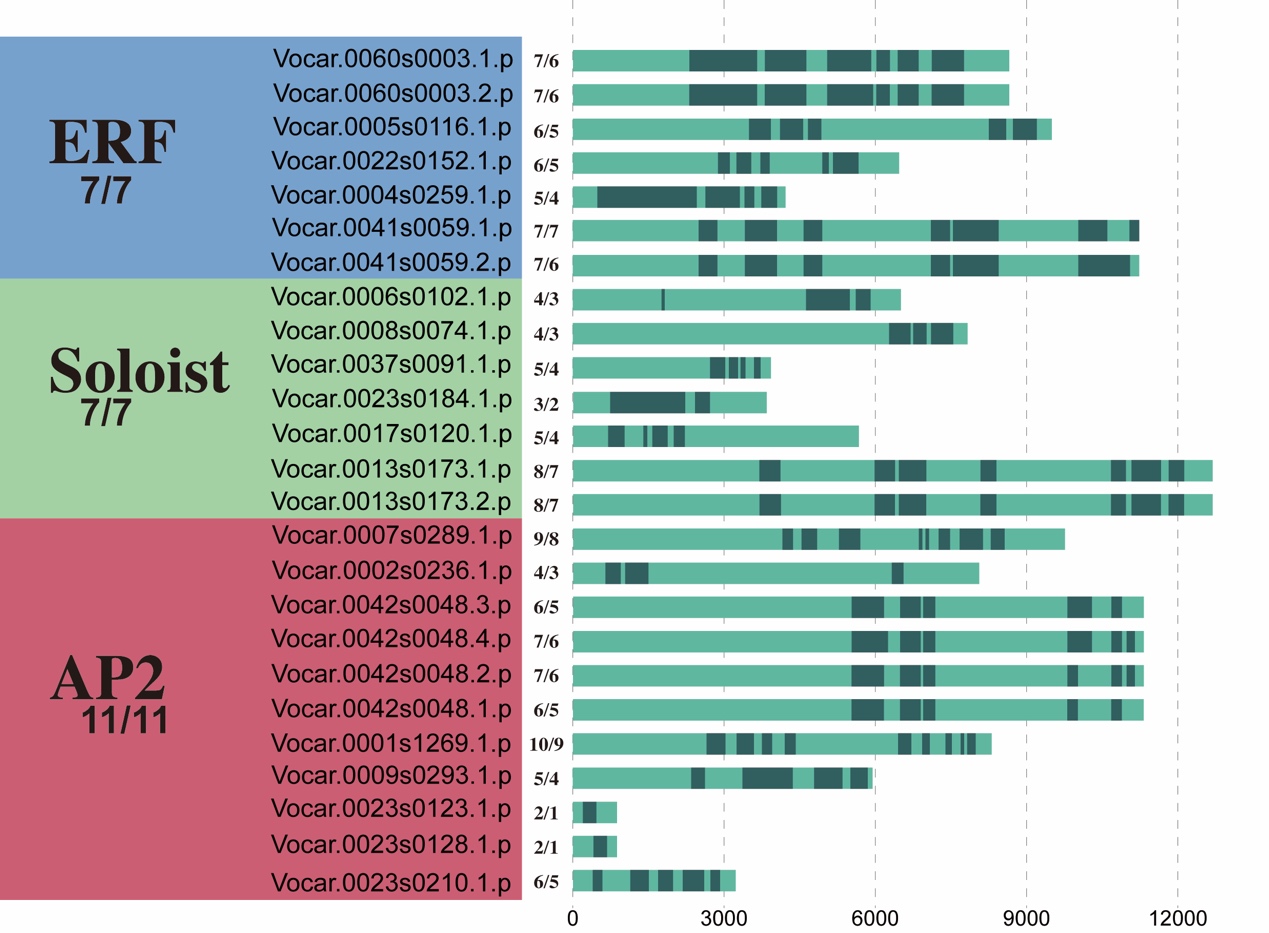


**FIGURE S6 | *AP2/ERF* genes structure in *V. carteri.*** Based on genome annotation file, the evolview website was used to show *VcAP2/ERF* gene structure. The red, pink, green, blue, and cyanine represent AP2, RAV, Soloist, ERF, and DREB, respectively. The light green and ink green was exon and intron, respectively. This ratio mean the proportion between members containing introns and their all subfamilies.


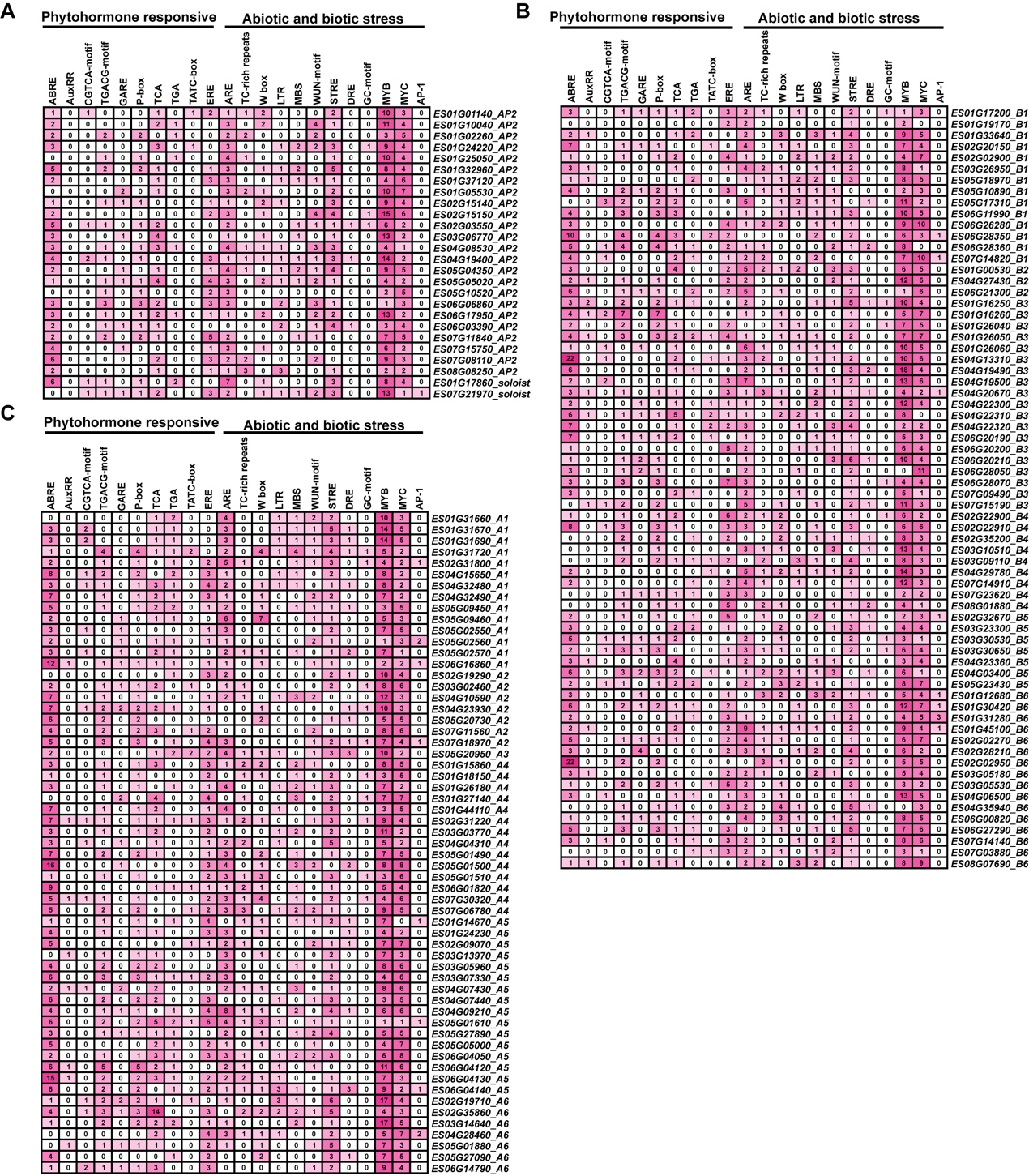


**Figure S7 | Cis-element analysis of the promoter regions of EsAP2/ERF genes.** Promoter of 3000 bp length of 153 *EsAP2/ERF* genes was used to predict cis-elment in PlantCARE. (A), (B) and (C) represent cis-element distribution on AP2 subfamily and Soloist subfamily, ERF subfamily and DREB subfamily. The quantity of cis-element were shown thought heatmap between red and white.


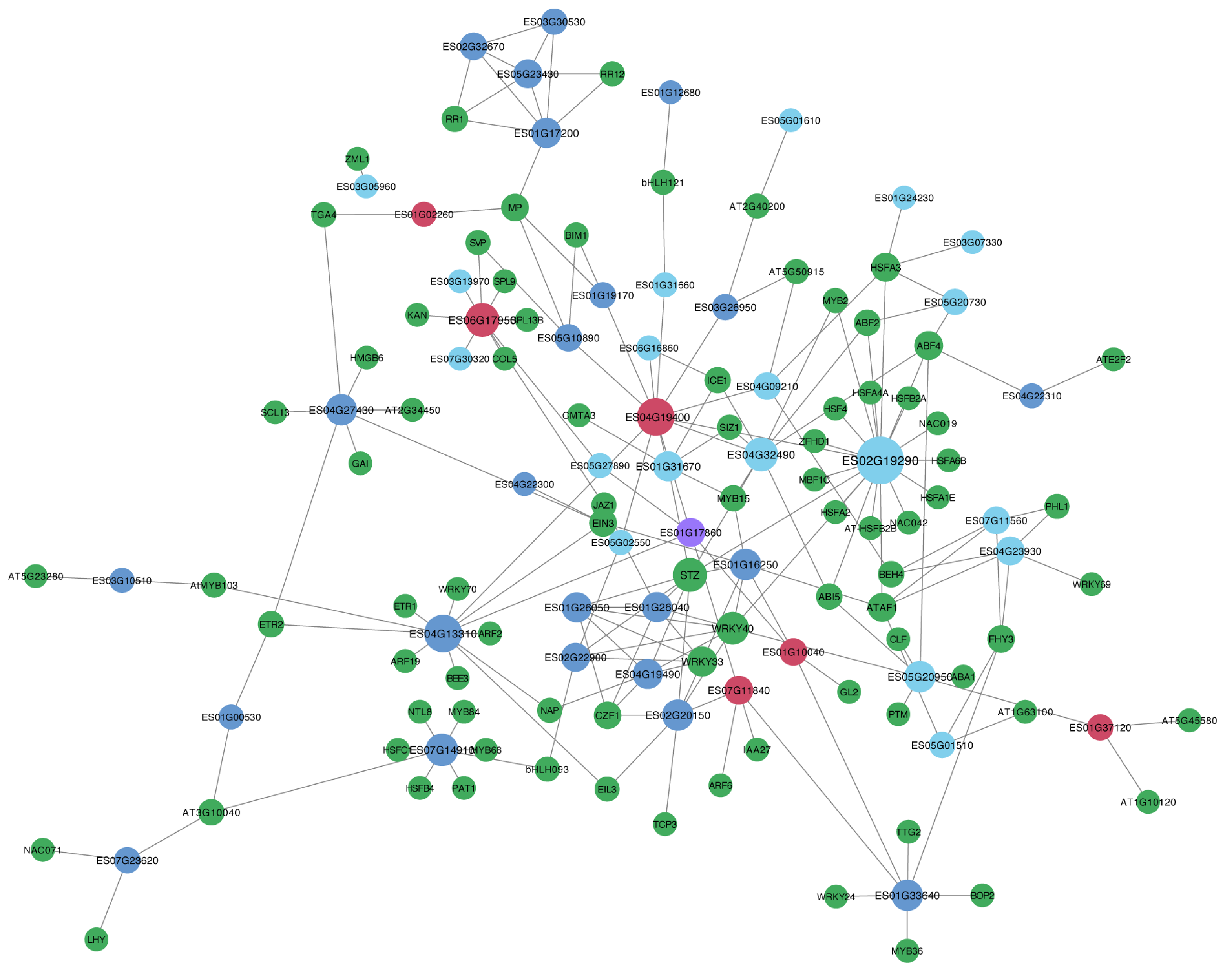


**Figure S8** | **Protein and protein interaction network assembly of EsAP2/ERF transcription factors.** The 49 EsAP2/ERF TFs and 160 other TFs were used to generate the protein network assembly network based on the String database.


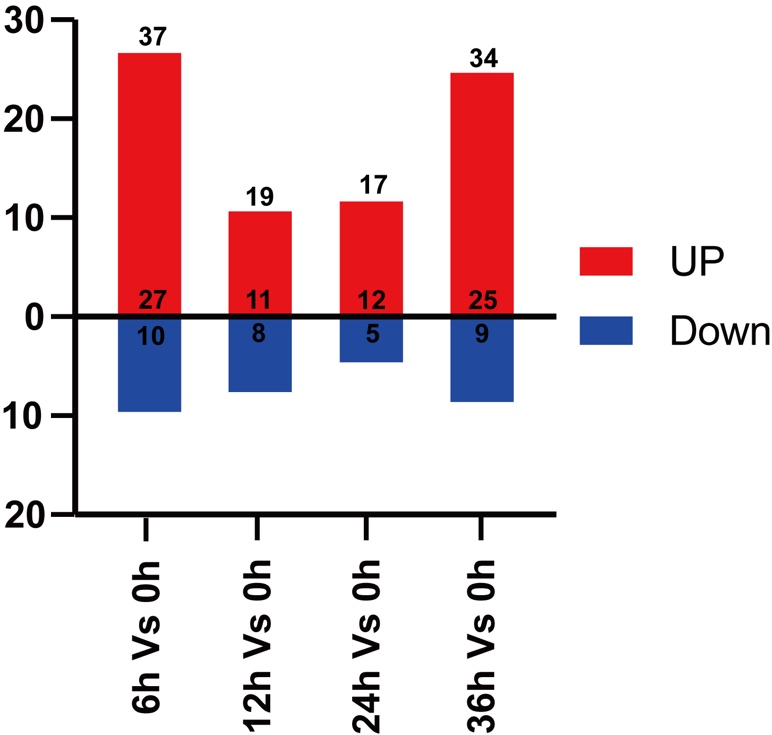


**Figure S9** | **The number of differentially expressed *EsAP2/ERF* genes at different time points after drought stress.** The red and blue columns represent up-regulated and down-regulated genes, respectively.
